# Public Risk-Taking and Rewards During the COVID-19 Pandemic - A Case Study of Remdesivir in the Context of Global Health Equity

**DOI:** 10.34172/ijhpm.2020.166

**Published:** 2020-09-06

**Authors:** Sabrina Wimmer, Sarai M. Keestra

**Affiliations:** ^1^University Medical Center Groningen, University of Groningen, Groningen, The Netherlands.; ^2^Harvey E. Beardmore Division of Pediatric Surgery, The Montreal Children’s Hospital, McGill University Health Centre, Montreal, QC, Canada.; ^3^Amsterdam UMC, University of Amsterdam, Amsterdam, the Netherlands.; ^4^Department of Global Health and Development, London School of Hygiene and Tropical Medicine, London, UK.

**Keywords:** Biomedical R&D, Innovation, Access, Public Value

## Abstract

Public investment, through both research grants and university funding, plays a crucial role in the research and development (R&D) of novel health technologies, including diagnostics, therapies, and vaccines, to address the coronavirus disease 2019 (COVID-19) pandemic. Using the example of remdesivir, one of the most promising COVID-19 treatments, this paper traces back public contributions to different stages of the innovation process. Applying the Risk-Reward Nexus framework to the R&D of remdesivir, we analyse the role of the public in risk-taking and reward and address inequities in the biomedical innovation system. We discuss the collective, cumulative and uncertain characteristics of innovation, highlighting the lack of transparency in the biomedical R&D system, the need for public investment in the innovation process, and the "time-lag" between risk-taking and reward. Despite the significant public transnational contributions to the R&D of remdesivir, the rewards are extracted by few actors and the return to the public in the form of equitable access and affordable pricing is limited. Beyond the necessity to treat remdesivir as a global public good, we argue that biomedical innovation needs to be viewed in the broader concept of public value to prevent the same equity issues currently seen in the COVID-19 pandemic. This requires the state to take a market-shaping rather than market-fixing role, thereby steering innovation, ensuring that patents do not hinder global equitable access and affordable pricing and safeguarding a global medicines supply.

## Background

 With 21.9 million confirmed coronavirus disease 2019 (COVID-19) cases at the time of writing^1^ (August 18, 2020), scientists across the world are partaking in a race to identify diagnostics, treatments and vaccines for severe acute respiratory syndrome coronavirus 2 (SARS-CoV-2). Many of these novel research initiatives are headed by public sector institutions, including universities and non-profit organisations, which lead nearly a third of vaccine development projects^2^. Public investments are a vital catalyst for accelerating the research and development (R&D) of new health technologies to address the coronavirus pandemic, as highlighted by Universities Allied for Essential Medicines’ public funding tracker.^3^

 The World Health Organization (WHO) defines health innovation in the context of biomedical R&D as a “new or improved” product that “improves people’s health and well-being.”^4^ COVID-19 has highlighted the underlying existing health disparities within our societies, with vulnerable groups being more susceptible to becoming infected or developing severe disease and dying.^5-7^ Due to existing inequities in the current pharmaceutical system regarding pricing, production capacity, and health system resources, issues with equitable access to health technologies for COVID-19 inevitably arise. The divide in access to novel treatments, diagnostics and vaccines will be especially poignant between higher- and lower-income countries and may deepen existing health disparities within countries themselves.

 In this paper we trace back public contributions to different stages of the R&D process of Gilead Sciences’ (Gilead) remdesivir, which is currently undergoing multiple clinical trials as a promising candidate treatment for SARS-CoV-2. Based on the R&D of other drugs, we hypothesise that public funding and research conducted at publicly-funded research institutions (PFRIs) played an important role in driving forward remdesivir’s R&D, whereas few actors would reap the benefits. In order to test this hypothesis, we systematically search the pre-pandemic scientific literature on publications on remdesivir, and analyse main sponsorship of clinical trials on remdesivir to approximate the public contributions into the R&D of this therapeutic.

 We introduce the Risk-Reward Nexus^8^ proposed by Lazonick and Mazzucato and apply this framework to biomedical innovation, using remdesivir as a case study. Analysing the relationship between innovation and inequity, we discuss the collective, cumulative and uncertain characteristics of innovation. Furthermore, we critically appraise the actors that contributed to the innovation process and the actors that appropriate returns from the innovation. Beyond looking at the inequities in risk and rewards in biomedical innovation, we discuss remdesivir in the context of global health equity meaning that “everyone should have a fair opportunity to achieve their full health potential,”^9^ which we argue should be an integral component of biomedical innovation during this pandemic and beyond. We thereby seek to create a framework that is applicable to other health innovations relevant to the COVID-19 pandemic and new health technologies more generally.

## Methods

###  Pre-pandemic Scientific Literature

 We systematically searched the pre-pandemic scientific literature on remdesivir from 2012, when its molecular structure was first published by Cho et al, until the end of 2019, when COVID-19 first emerged. For this purpose we searched PubMed, Ovid, and Google Scholar for primary research studies published in English on “remdesivir” or “GS-5734” (see Supplementary file 1 discloses the complete search strategies used in this paper). Additionally, we asked colleagues for studies not identified by our original search and used snowballing to look for further references missed. Searches were conducted between the 6th and 18th of July.

 From the papers identified we extracted the affiliation of the first and final author, categorising these affiliations as “Gilead,” “other private actor,” or as being a PFRI. For the purpose of this paper we defined PFRIs as including universities, hospitals, government agencies, research funding bodies, charities, or any other institution that receives public funds or relies on donations from the public. From the complete author list we calculated how many of the total affiliations fell into these three categories as well. In these calculations we took into account that some authors list more than one affiliation, and in such instances we weighted both affiliations equally. We also compiled a list of PFRI affiliations and the countries in which these PFRIs are based.

###  Grant Information

 Next, we extracted the different public funding bodies that contributed to the study from the acknowledgement section of identified articles. Where possible, we obtained grant numbers and traced these back to the original funding bodies to list the amount and purpose of the grant. We supplemented our findings with information from briefings by the US-based non-for profit organisations Knowledge Ecology International^10^ and Public Citizen,^11^ which previously investigated the public contributions towards remdesivir’s R&D process.

###  Clinical Trial Sponsorship

 After assessing academic publications on remdesivir that preceded the COVID-19 pandemic, we also systematically searched for clinical trials on remdesivir registered on ClinicalTrials.gov. All registered trials listing remdesivir or GS-5734 as the primary intervention were included and the main trial sponsor was categorised as “Gilead,” “other private actor,” or “PFRI.” Next, we disseminated whether trials sponsored by PFRIs were conducted by a university, hospital, charity, or government agency. We put university hospitals in the category “hospital.” We also noted the countries in which these PFRIs were based. Both interventional trials and observational studies were included to capture both trials studying the efficacy as well as the safety of the drug. Firstly, we searched through all trials mentioning “remdesivir” or “GS-5734,” the molecular compound name of remdesivir. Additionally, an advanced search with “interventional (clinical) trials” or “observational trials” as filters was conducted, for trials in which “remdesivir” was used as one of the main interventions. We excluded studies listing remdesivir as a part of the standard of care of the control group, as these trials do not directly study the clinical efficacy or safety of the drug. The search was conducted on July 17, 2020.

## Results

###  Pre-pandemic Scientific Literature 

 Our systematic search of the pre-pandemic scientific literature through PubMed, Ovid, and Google Scholar identified 14 articles published between 2012-2019 on remdesivir or GS-5734 (see Supplementary file 1). Of the 14 publications, only two listed Gilead as the primary affiliation for the first and final author (Table 1). Apart from the first publication that identified the molecular structure of remdesivir, which was authored by in-house scientists of Gilead, PFRI-affiliations on the author list ranged from 15%-100%, with on average more than half of the author affiliations being linked to a PFRI (64% ± 27% (SD)). Researchers involved in the early research on remdesivir were affiliated with PFRIs in the United States, the United Kingdom, Poland, Belgium, Guinea, Canada, the Democratic Republic of the Congo (DRC), Senegal, France, as well as the WHO, which contributed to the first clinical trial of remdesivir in humans.

**Table 1:** The Contribution of Publicly Funded Research Institutions to Publications on Remdesivir (GS-5734) Between 2012-2019

**Publication Title**	**Year**	**First Author**	**First Author Affiliation**	**Final Author**	**Final Author Affiliation**	**Specification Authors Affiliation PFRI** ^a^	**Percentage Affiliations PFRI**
Synthesis and antiviral activity of a series of 10-substituted 4-aza-7,9-dideazaadenosine C-nucleosides	2012	Cho, Aesop^12^	Gilead	Kim, Choung U.	Gilead	NA	0
Therapeutic efficacy of the small molecule GS-5734 against Ebola virus in rhesus monkeys	2016	Warren, Travis K.^13^	USAMRIID	Bavari, Sina	USAMRIID	USAMRIID; Therapeutic Development Center; CDC; Boston University School of Medicine, Boston	57%
Late Ebola virus relapse causing meningoencephalitis: a case report	2016	Jacobs, Michael^14^	Royal Free London NHS Foundation Trust	Thomson, Emma C	Royal Free London NHS Foundation Trust	Royal Free London NHS Foundation Trust; University College London; Edinburgh Royal Infirmary; Public Health England; Queen Elizabeth University Hospital Glasgow; MRC–University of Glasgow Centre for Virus Research, Glasgow, UK	100%
Broad-spectrum antiviral GS-5734 inhibits both epidemic and zoonotic coronaviruses	2017	Sheahan, Timothy P.^15^	UNCCH	Baric, Ralph S.	UNCCH	UNCCH; VUMC; Jagiellonian University	43%
GS-5734 and its parent nucleoside analog inhibit Filo, Pneumo-, and Paramyxoviruses	2017	Lo, Michael K.^16^	CDC	Spiropoulou, Christina F.	CDC	CDC	41%
First newborn baby to receive experimental therapies survives Ebola virus disease	2017	Dörnemann, Jenny^17^	Médecins Sans Frontières	Antierens, Annick	Médecins Sans Frontières	Médecins Sans Frontières Belgium and Guinea; Institute of Tropical Medicine Antwerp; CDC; Emory University	100%
Discovery and synthesis of a phosphoramidate prodrug of a Pyrrolo[2,1-f][triazin-4-amino] Adenine C-Nucleoside (GS-5734) for the treatment of Ebola and emerging viruses	2017	Siegel, Dustin^18^	Gilead	Mack-man, Richard L.	Gilead	USAMRIID; University of California; CDC	15%
Coronavirus susceptibility to the antiviral remdesivir (GS-5734) is mediated by the viral polymerase and the proofreading exoribonuclease	2018	Agostini, Maria L.^19^	VUMC	Denison, Mark R.	VUMC	VUMC; UNCCH; University of the South	63%
Initiation, extension, and termination of RNA synthesis by a paramyxovirus polymerase	2018	Jordan, Paul C.^20^	Allos BioPharma	Deval, Jerome	Allos BioPharma	CDC	25%
Remdesivir (GS-5734) protects African green monkeys from Nipah virus challenge	2018	Lo, Michael K.^21^	CDC	Wit, Emmie De	NIAID	CDC; NIAID	79%
A randomized, controlled trial of Ebola virus disease therapeutics	2019	Mulangu, Sabue^22^	Institut National de Recherche Biomédicale Democratic Republic of Congo	Nordwall, Jacqueline	University of Minnesota	Institut National de Recherche Biomédicale; International Medical Corps; Médecins sans Frontières; the World Health Organisation; the Alliance for International Medical Action; the Biomedical Advanced Research and Development Authority; NIAID; University of Minnesota	75%
Mechanism of inhibition of Ebola virus RNA-dependent RNA polymerase by remdesivir	2019	Tchesnokov, Egor P.^23^	University of Alberta	Götte, Matthias	University of Alberta	University of Alberta & the Li Ka Shing Institute of Virology at University of Alberta	67%
Characterization of Ebola virus from an ongoing outbreak in Ituri and North Kivu, DR Congo to guide to response activities	2019	McMullan, Laura K.^24^	CDC	Albarino, Cesar	CDC	CDC	89%
Broad spectrum antiviral remdesivir inhibits human endemic and zoonotic deltacoronaviruses with a highly divergent RNA dependent RNA polymerase	2019	Brown, Ariane J.^25^	UNCCH	Sheahan, Timothy P	UNCCH	UNCCH; VUMC	80%

Abbreviations: NHS, National Health Service; PFRI, publicly funded research institution; USAMRIID, United States Army Medical Research Institute of Infectious Diseases; UNCCH, University of North Carolina at Chapel Hill; CDC, Centers for Disease Control and Prevention; VUMC, Vanderbilt University Medical Center; NIAID, National Institute of Allergy and Infectious Diseases. ^a^For the purpose of this paper we defined PFRIs as including universities, hospitals, government agencies, research funding bodies, or any other institution that receives public funds as well as charities relying on donations from the public.

###  Grant Information

 We identified at least 20 different sources of public funding into remdesivir’s R&D through research grants, academic fellowships, or core funding from government agencies (Table 2 and Supplementary file 2). Except for one article which did not disclose its funding sources (Dörnemann et al^17^), all studies published after the original remdesivir study by Cho et al acknowledged at least one source of public funding. Although the Centres for Disease Control and Prevention (CDC) and the National Institutes of Health (NIH) funded most of the research into remdesivir, grants from the United Kingdom and Canadian funding bodies also played a role in the public investment into the R&D process of this drug.

**Table 2:** Public Funding Into the Pre-Pandemic R&D of Remdesivir

**Grant or Project Name **	**Amount (If Available)**	**PFRIs Involved**	**Relevant Publication(s)**	**Direct Citation From Article**
Core Funding	Unknown	CDC	Siegel et al^18^; Lo et al^16^; Jordan et al^20^; McMullan et al^24^	Siegel et al^18^: "CDC core funding supported the work done by M.K.L. at CDC"; Lo et al^16^: "This work was funded by CDC core funding"; Jordan et al^20^: "CDC core funding supported MKL and CFS [two co-authors from the CDC]"; Lo et al^21^: "This work is supported by (…) core funding at CDC (to M.K.L., J.M.G., N.R.P., J.D.K., S.T.N., S.R.Z., and C.F.S.)"; McMullan et al^24^: "Funding: US CDC."
Core Funding	Unknown	USMRAIID	Siegel et al^18^	"These studies were in part supported by the JSTO-CBD of the DTRA under Plan No. CB10218." In the article it is mentioned that: "The partnership with government organizations, including CDC and USAMRIID, that generated the screening data and conducted the rhesus efficacy studies was critical to the successful identification of [remdesivir].”
CB10218	Unknown	JSTO-CBD DTRA	Warren et al^13^	"Studies at USAMRIID were in part supported by the JSTO-CBD of the DTRA under plan #CB10218."
R01AI113321	$1 659 997	NIH, Boston University	Warren et al^13^	"Work in the Fearns laboratory was supported by NIH R01AI113321."
Core Funding	Unknown	Royal Free London NHS Foundation Trust	Jacobs et al^14^	"The work was funded by the Royal Free London NHS Foundation Trust, without external grants."
Core Funding	Unknown	Medical Research Council UK	Jacobs et al^14^	"Sequencing and bioinformatics analyses were funded by the Medical Research Council."
102789/Z/13/Z	Unknown	Wellcome Trust, MRC–University of Glasgow Centre for Virus Research	Jacobs et al^14^	"E[mma] C. T[hompson] [the final author of the paper] is funded by the Wellcome Trust (grant number 102789/Z/13/Z)."
R01 AI132178	$3 788 580	NIH, UNC-Chapel Hill	Brown et al^25^	"We would like to acknowledge the following funding sources (...) and a partnership grant from the NIH, United States (5R01AI132178).”
R01 AI108197	$4 235 454	NIH, Vanderbilt University	Sheahan et al^15^; Agostini et al^19^	Sheahan et al: "Grants from the NIH AI108197"; Agostini et al: "This work was supported by the NIH grants R01AI108197."
U19 AI109761	$32 615 934	NIH, Columbia University	Sheahan et al^15^	Sheahan et al: "Grants from the NIH (...) AI109761."
P30 DK065988	Unknown	NIH, UNC- Chapel Hill	Sheahan et al^15^; Agostini et al^19^	Sheahan et al: "Cystic Fibrosis and Pulmonary Research and Treatment Center(…) and NIH P30DK065988)"; Agostini et al: "UNC Cystic Fibrosis and Pulmonary Diseases Research and Treatment Center (…) NIH P30DK065988."
BOUCHE15RO	Unknown	UNC-Chapel Hill Cystic Fibrosis and Pulmonary Research Treatment Center	Sheahan et al^15^; Agostini et al^19^	Sheahan et al: "Cystic Fibrosis and Pulmonary Research and Treatment Center BOUCHE15RO"; Agostini et al: "the UNC Cystic Fibrosis and Pulmonary Diseases Research and Treatment Center BOUCHE15RO."
U19 AI109680	$34 907 030	NIH, University of Alabama, UNC-Chapel Hill, and VUMC	Sheahan et al^15^; Agostini et al^19^; Brown et al^25^	Sheahan et al: "Antiviral Drug Discovery and Development Center (5U19AI109680)"; Agostini et al: "This work was supported by the Antiviral Drug Discovery and Development Center 5U19AI109680"; Brown et al: "We would like to acknowledge the following funding sources, Antiviral Drug Discovery and Development Center (5U19AI109680)."
5T32AI089554	$726 584	NIH, Vanderbilt University	Agostini et al^19^	"This work was supported by (…) NIH grants (…) 5T32AI089554 (M.L.A.)."
T32 AI007419	Unknown	NIH, UNC-Chapel Hill	Brown et al^25^	"KD was supported by a fellowship from the NIH NIAID virology training grant (T32 AI007419)."
Postdoctoral Fellowship	Unknown	American Society for Microbiology Postdoctoral Fellowship, CDC	Lo et al^16^	"Anne L. Hotard is supported by an American Society for Microbiology Postdoctoral Fellowship."
Intramural Research Program	Unknown	NIAID, NIH	Lo et al^21^	"This work is supported by the Intramural Research Program of NIAID, NIH (to F.F., J.C., H.F., and E.d.W.)."
Clinical Trial Funding	Unknown	NIAID, NIH	Mulangu et al^22^	"Supported primarily by the NIAID, NIH."
HHSN261200800001E	Unknown	National Cancer Institute	Mulangu et al^22^	"Some funding for NIAID was provided by the National Cancer Institute through a contract (HHSN261200800001E) with Leidos Biomedical Research and subcontracts to the Mitchell Group."
CIHR159507	Unknown	Canadian Institutes of Health Research	Tchesnokov et al^23^	"This research was funded by grants from the CIHR (grant number 159507)."

Abbreviations: R&D, Research and development; NHS, National Health Service; PFRI, publicly funded research institution; USAMRIID, United States Army Medical Research Institute of Infectious Diseases; CDC, Centers for Disease Control and Prevention; CIHR, Canadian Institutes of Health Research; JSTO-CBD, The Joint Science and Technology Office for Chemical and Biological Defense; DTRA, Defense Threat Reduction Agency; NIAID, National Institute of Allergy and Infectious Disease; NIH: National Institutes of Health; VUMC, Vanderbilt University Medical Center.

###  Clinical Trial Sponsorship

 We identified 24 relevant clinical trials registered on ClinicalTrials.gov using remdesivir as an intervention or studying its safety (Table 3; see Supplementary 3 for full search results, recruitment status, phase of trial and URL to the respective clinical trials). Of the 24 clinical trials registered, 4 were sponsored by “Gilead,” 2 by “other private actors” (ViralClear Pharmaceuticals, Inc. and Hoffman-La Roche) and 18 by “PFRIs.” Within the category of PFRIs sponsored trials, 7 were sponsored by (university) hospitals or health centres, 4 by universities, 6 by government agencies, and 1 by a charity. These PFRIs were located across the United States, China, Uganda, France, Switzerland, Norway, and Canada.

**Table 3:** Clinical Trial Sponsorship of 24 Clinical Trials on Remdesivir (GS-5734), and the Contribution of Publicly Funded Research Institutions Therein

**ClinicalTrials.gov Identifier**	**Name Trial**	**Main Sponsor**	**Sponsor Category **	**Type of PFRI** ^a^	**Country That PFRI Is Based in**
NCT04257656	A trial of remdesivir in adults with severe COVID-19	Capital Medical University	PFRI	University	China
NCT04385719	Drug-drug interactions between remdesivir and commonly used antiretroviral therapy (RemTLAR)	Makerere University	PFRI	University	Uganda
NCT04252664	A trial of remdesivir in adults with mild and moderate COVID-19	Capital Medical University	PFRI	University	China
NCT04431453	Study to evaluate the safety, tolerability, pharmacokinetics, and efficacy of remdesivir (GS-5734^TM^) in participants from birth to <18 years of age with coronavirus disease 2019 (COVID-19) (CARAVAN)	Gilead Sciences	Gilead Sciences	NA	NA
NCT04302766	Expanded access remdesivir (RDV; GS-5734^TM^)	U.S. Army Medical Research and Development Command	PFRI	Government agency	US
NCT04292899	Study to evaluate the safety and antiviral activity of remdesivir (GS-5734^TM^) in Participants with severe coronavirus disease (COVID-19)	Gilead Sciences	Gilead Sciences	NA	NA
NCT04365725	Multicenter, retrospective study of the effects of remdesivir in the treatment of severe COVID-19 infections (REMDECO-19)	Assistance Publique - Hôpitaux de Paris	PFRI	Hospital	France
NCT04292730	Study to evaluate the safety and antiviral activity of remdesivir (GS-5734^TM^) in Participants with moderate coronavirus disease (COVID-19) compared to standard of care treatment	Gilead Sciences	Gilead Sciences	NA	NA
NCT04323761	Expanded access treatment protocol: remdesivir (RDV; GS-5734) for the treatment of SARS-CoV2 (CoV) infection (COVID-19)	Gilead Sciences	Gilead Sciences	NA	NA
NCT04410354	Study of merimepodib in combination with remdesivir in adult patients with advanced COVID-19	ViralClear Pharmaceuticals, Inc.	Other private actor	NA	NA
NCT04409262	A study to evaluate the efficacy and safety of remdesivir plus tocilizumab compared with remdesivir plus placebo in hospitalized participants with severe COVID-19 pneumonia (REMDACTA)	Hoffmann-La Roche	Other private actor	NA	NA
NCT02818582	GS-5734 to assess the antiviral activity, longer-term clearance of Ebola virus, and safety in male Ebola survivors with evidence of Ebola virus persistence in semen	NIAID	PFRI	Government agency	US
NCT04401579	Adaptive COVID-19 Treatment Trial 2 (ACTT-2)	NIAID	PFRI	Government agency	US
NCT04330690	Treatments for COVID-19: Canadian Arm of the SOLIDARITY Trial (CATCO)	Sunnybrook Health Sciences Centre	PFRI	Hospital	Canada
NCT04280705	Adaptive COVID-19 Treatment Trial (ACTT)	NIAID	PFRI	Government agency	US
NCT04321616	The efficacy of different anti-viral drugs in COVID-19 infected patients	Oslo University Hospital	PFRI	Hospital	Norway
NCT03719586	Investigational therapeutics for the treatment of people with Ebola virus disease	NIAID	PFRI	Government agency	US
NCT04315948	Trial of Treatments for COVID-19 in Hospitalized Adults (DisCoVeRy)	Institut National de la Santé Et de la Recherche Médicale, France	PFRI	Government agency	France
NCT04314817	Adverse Events Related to Treatments Used Against Coronavirus Disease 2019 (CovidTox)	Groupe Hospitalier Pitie-Salpetriere	PFRI	Hospital	France
NCT04365764	Effect of treatments in patients hospitalized for severe COVID-19 pneumonia: a multicenter cohort study	Groupe Hospitalier Pitie-Salpetriere	PFRI	Hospital	France
NCT04356417	Long-term use of drugs that could prevent the risk of serious COVID-19 infections or make it worse (TRAPSAH)	Assistance Publique - Hôpitaux de Paris	PFRI	Hospital	France
NCT04349410	The Fleming [FMTVDM] Directed COVID-19 Treatment Protocol (FMTVDM)	The Camelot Foundation	PFRI	Charity	US
NCT04278404	Pharmacokinetics, pharmacodynamics, and safety profile of understudied drugs administered to children per standard of care (POPS or POP02)	Duke University	PFRI	University	US
NCT04351503	A systems approach to predict the outcome of SARS-CoV-2 in the population of a city; COVID-19	University Hospital, Basel, Switzerland	PFRI	Hospital	Switzerland

Abbreviations: PFRI, publicly funded research institution; NIAID, National Institute of Allergy and Infectious Disease; COVID-19, coronavirus disease 2019; SARS-CoV-2, severe acute respiratory syndrome coronavirus 2. ^a^PFRI is an acronym for publicly-funded research institution. For the purpose of this paper we defined PFRIs as including universities, hospitals, government agencies, research funding bodies, or any other institution that receives public funding as well as charities relying on donations from the public.

## Discussion

###  The Risk-Reward Nexus Applied to Remdesivir

####  The Collective, Cumulative and Uncertain Characteristics of Biomedical Innovation 

 The Risk-Reward Nexus was first proposed by Lazonick and Mazzucato^8^ and considers innovation as a collective, cumulative, and uncertain process. Here we apply these characteristics to the R&D of remdesivir.

 As the remdesivir case study illustrates, biomedical innovation is collective, meaning it requires the skills and efforts of people from different backgrounds and often involves a network of institutions.^8^ These collective contributions may be in the form of knowledge (eg, information sharing), human resources (eg, scientists, clinical trial participants), infrastructure (eg, research facilities, hospitals), and financial resources (eg, governments grants). In 2012, Gilead-affiliated authors published the first study about the new compound GS-5734 alongside several other compounds that showed broad spectrum antiviral activity. In 2014, in the midst of the Ebola outbreak, a research initiative hosted by the University of Alabama at Birmingham received a large National Institute of Allergy and Infectious Disease (NIAID) grant to initiate safety and efficacy studies of remdesivir along other antivirals against high-priority emerging infections.^26^ Vanderbilt University, the University of North Carolina at Chapel Hill (UNCCH) and the University of Alabama at Birmingham conducted several studies looking at remdesivir’s activity against betacoronavirae, such as the severe acute respiratory syndrome (SARS) and the Middle East respiratory syndrome (MERS).^15,19,25^ A collaboration with the CDC and the United States Army Medical Research Institute of Infectious Diseases (USAMRIID) screened Gilead’s compound library for potential Ebola virus treatments and tested remdesivir against RNA viruses.^18^ Before the COVID-19 pandemic, the NIH sponsored two clinical trials in humans exploring remdesivir’s antiviral activity against Ebola. The larger of these was funded and sponsored by the NIAID and the NIH, and further supported by the African Coalition for Epidemic Research, Response, and Training and the government of the DRC. The WHO led the trial logistics, while Gilead provided the drug for comparison with other promising Ebola virus therapies.^10^ In this study, we identified that more than half of the authors of the pre-pandemic scientific literature were affiliated with PFRIs in 9 countries and the WHO. Publicly sponsored clinical trials for COVID-19 are currently underway in at least 7 countries, and involve a wide variety of actors, including (university) hospitals or health centres, universities, government agencies, and a charity. Thus, significant institutional support from public actors, both in terms of funding as well as logistics and research, pushed remdesivir along the development pipeline.

 The remdesivir case study highlights that biomedical innovation is cumulative, meaning that innovation that originated earlier is being used in the development of a new innovation or further developed.^8^ Remdesivir was originally developed as a broad spectrum antiviral,^12^ building on the ProTide technology developed at the University of Cardiff in the 1990s.^27^ It was then undergoing safety and efficacy studies against high-priority infections and found to be effective against RNA viruses, suggesting that it could be developed as a broad-spectrum antiviral against coronaviruses.^19^ Between 2018 and 2019, intravenous application of remdesivir was studied for Ebola in the DRC, where it was found to be adequately tolerated but less effective compared to other therapies.^22^ Based on this previously gained knowledge, remdesivir was first given to COVID-19 patients on a compassionate-use basis and is currently undergoing at least 24 interventional clinical trials for COVID-19 disease.^28^

 The remdesivir case study shows that biomedical innovation is uncertain, meaning that there is neither a guarantee that investments into R&D will actually result in innovation, nor a guaranteed return, whether financially or in the form of equitable access.^8^ Remdesivir’s initial development as an antiviral against hepatitis C is an example for the high-risk of early research, with many compounds not making it further than the early R&D stages.^29^ US government investment was necessary to identify and repurpose the compound for application in Ebola virus disease, which had limited potential for financial return for Gilead Sciences. After other treatments were found to be superior to remdesivir against Ebola virus disease, the compound was shelved again until COVID-19 emerged.^30^ Early results showed some likely clinical benefits in certain patient groups,^31,32^ which led the US Food and Drug Administration (FDA) to issue an emergency use authorization for severe COVID-19 disease.^33^ Multiple clinical trials are currently comparing remdesivir to placebo and other treatments. Dexamethasone, a cheap and widely available drug compared to remdesivir, showed to reduce mortality in hospitalised patients.^34^ Further clinical trials are needed to establish the effectiveness of remdesivir compared to dexamethasone and other competitors to determine whether remdesivir will become a treatment of choice for COVID-19, which highlights yet again the uncertainty of biomedical innovation.

###  Equity in Public Risk-Taking and Reward 

 After focusing on the collective, cumulative and uncertain characteristics of the innovation process of remdesivir, we assess the equity aspect of the Risk-Reward Nexus, hereby applying a global health equity focus. We address herein the following questions: who has taken the risks, who gets the rewards, and is the degree of the rewards proportional to the risks taken?

 Who are the actors that invested labor and capital in the R&D of remdesivir? The innovative compound, GS-5734, was developed by the US-based biopharmaceutical company Gilead Sciences, applying amongst other processes and methods the ProTide technology developed at the University of Cardiff, UK, which received significant amounts of European public funding at the time. For the further R&D of remdesivir pre-pandemic, this study identified at least 20 different sources of public funding, including research grants, academic fellowships, and core funding from government agencies. The amount of funding was traceable for 6 of these 20 public funding grants, totalling nearly US$78 million, which is likely an underreporting of the total public contribution. The NIH and CDC were the main public funders observed in this study, supported by PFRIs and public funders from at least 3 countries. Additionally, the US Department of Defense collaborated with Gilead through the signing of an Other Transaction Agreement with a total contract value of nearly $50 million not captured in our findings.^35,36^ We recorded 24 registered clinical trials, of which 6 were sponsored by private actors and 18 by PFRIs located in at least 7 countries. This shows that public actors from multiple countries played a crucial role along remdesivir’s R&D pipeline, and continue to do so in the clinical trial stages. Our observation is supported by the findings that all 210 new molecular entities approved by the FDA between 2010 and 2016 received NIH funding.^37^ Furthermore, public funding played a major role in the late stage development of at least 25% of drugs approved by the FDA between 2008 and 2017.^38^ Overall, total biomedical R&D expenditure in 2012 amounted to US$81.8 billion in Europe and US$119.3 billion in the United States, of which the public sector directly contributed US$28.1 billion and US$48.9 billion respectively, equivalent to 30%-35% of biomedical R&D.^39^ Finally, the public sector is a main funder of high-risk, early research,^40^ making public investments especially important for priority research areas where, despite significant health need, a lack of private sector investment prevails.^41^

 Which actors ultimately benefit from remdesivir as a biomedical innovation? In our current biopharmaceutical system, the owner of the key patent rights (this may be the owner of a patent or a licensee) has the main control over the extraction of rewards. This means they hold decision power regarding pricing, production, and reimbursement agreements with governments and health systems across the world. The provisional patent application (61/047263) claiming the remdesivir compound formula lists two Gilead in-house scientists as co-inventors.^10^ In October 2015, Gilead Sciences affiliated scientists filed a patent application (62/239696) associated with remdesivir describing “*Methods for Treating Arenaviridae and Coronaviridae Virus Infections.*”^10^ Shortly after, researchers largely affiliated with PFRIs announced the “*discovery of a novel small molecule GS-5734*” against Ebola virus in a *Nature* publication.^13^ A study on the impact of public grant funding on patenting by pharmaceutical and biotechnology firms has shown that NIH funding increases private-sector patenting, with every additional $10 million in NIH funding generating 2.3 additional patents owned by the private sector.^42^

 In March 2020, Gilead applied for “orphan status” for remdesivir in the United States, which grants significant tax credits and reduced market approval fees. These financial incentives were created to foster investment into rare diseases, however, COVID-19 was anything but rare at the time and after public outcry Gilead rescinded its application.^43^ According to estimations by researchers affiliated with PFRIs, the production price of remdesivir is merely $0.93 per day of treatment.^44^ In May 2020, Gilead granted non-exclusive voluntary licenses to five generic manufacturers to distribute remdesivir to 127 countries for the duration of the pandemic.^45^ However, this agreement excluded 3.7 billion people and still leaves open the question how heavily affected countries not included in the list, such as Brazil, Peru, Mexico, China, and Russia can access and afford remdesivir. After donating the remaining stockpile of remdesivir to the US government,^46^ Gilead set the price for governments of developed countries at $390 per vial, or $2,340 for a 5-day treatment course, and at $520, or $3120, respectively, for US private insurance companies.^47^ In comparison, the Indian generic drug manufacturer Cipla Ltd priced its generic version of remdesivir at $53.34 per vial.^48^ The US Department of Health and Human Services secured more than 500 000 treatment courses, representing 100% of Gilead’s production in July, and 90% in August and September 2020.^49^ This agreement and the limited global production capacity will leave most of the world’s population without access to remdesivir, in addition to the strain on health systems caused by the high prices. Despite the global efforts in the R&D process highlighted above, the remdesivir case study demonstrates that rewards are extracted by few actors involved.

###  Risk and Reward From a Global Health Equity Perspective

 After establishing the extent of public contributions in the R&D of remdesivir and the limited public returns, we question the proportionality of the risks and rewards of remdesivir from a global health equity perspective. The WHO definition of equity in health encompasses two aspects. Firstly, equity in health, or health status, means “the attainment by all citizens of the highest possible level of physical, psychological and social well-being.”^50^ A large majority of the global population will not be able to access remdesivir when falling ill with COVID-19, denying them the fair opportunity to achieve their full health potential. The public risk taking and contributions in the innovation process, which included a significant number of patients across multiple countries (including the DRC, China, and the United Kingdom) who voluntarily risked their health by participating in clinical trials to advance science, should be reflected in the rewards, meaning global equitable and affordable access for all. However, as discussed above, this is currently not the case. The collective characteristic of innovation makes measuring the specific contributions of the various actors (public and private) difficult, further hindered by a lack of transparency in the biomedical R&D system. Private actors such as Gilead extract significantly more rewards, while incurring high costs for health systems and limiting access for patients. The uncertain characteristic of innovation is reflected in the need for public investment especially in early, high-risk research, and financial incentives for the private sector. The cumulative characteristic generates a “time-lag” between risk-taking and return, making public contributions especially at earlier stages less visible.

 Secondly, equity in healthcare means “healthcare resources are allocated according to need.”^50^ This requires an understanding of who the most vulnerable people are in our global society, for instance through vulnerability mapping on national, regional and global levels,^51^ and the willingness to address these inequities by providing treatment to those with the greatest need first. Yet, in the current pharmaceutical system, ability and willingness to pay are the main definer of who will be able to realise their need.^50^ Despite the collective involvement of many actors, both public and private, from high-, middle- and low-income countries across the globe in the R&D of remdesivir, only privileged few patients, primarily from high-income countries with strong health systems will be able to ultimately access the treatment.

 The Risk-Reward Nexus showcases a complex involvement of public and private actors in the biomedical innovation process. Currently, public investment in biomedical R&D is justified as remedying market failure where the private sector is underinvesting, such as high-risk early research or research areas with limited financial return. However, this approach of public sector risk-taking for private sector rewards of biomedical innovation is unproportional and further increases inequities in health and healthcare. The COVID-19 pandemic has underlined the need for making biomedical innovation a global public good as proposed by the Lancet Commission on Investing in Health.^52^ Simultaneously, the Risk-Reward Nexus shows that the current definition of global public goods as non-rivalrous and non-excludable (yet, treating one person reduces the quantity available and access can be prevented^53^) is restrictive and does not account for the complexity and nuances of socialised public risk-taking, neither does it offer solutions for extracting public rewards. The public (state) thus needs to take a more pro-active market-shaping rather than market-fixing role, thereby steering innovation, ensuring that patents do not hinder global equitable access and affordable pricing, and safeguarding a global medicines supply.^54^ This requires biomedical innovation to be viewed in the broader concept of public value rather than global public goods to prevent similar equity issues in the future as currently seen in the COVID-19 pandemic and highlighted in the remdesivir case study. Further research is necessitated on the setting of priorities to address (health) inequities, the establishment of decision-making processes that are inclusive and the need for focusing on outcomes of research policies and priorities.^55,56^ Lastly, a framework is needed that allows for a public return which, beyond focusing on financial return, emphasises health equity through global equitable access and affordable pricing.

 Due to the explorative nature of the methods of this study, our approach towards assessing the public contributions to different parts of remdesivir’s R&D has a number of limitations. The restriction of the systematic literature search to the pre-pandemic literature did not capture all novel research published on remdesivir and its efficacy against coronaviruses since the COVID-19 pandemic, thereby underestimating the public contributions and risk-taking in recent months. By recording the clinical trials on remdesivir we tried to capture later stage research as well. Secondly, it was not possible to specify exact amounts for all public funding grants due to a lack of transparency of grant databases, and the difficulty of tracing back exact amounts even when grant numbers were listed. Consequently, we likely underestimated the total contribution of public funds into remdesivir’s R&D. A final limitation to our approach is that conducting a systematic literature search focusing on remdesivir failed to capture the fundamental research into molecular chemistry that led to the methods and processes by which compound GS-5734 was created and the role of public funding therein. An important example is the ProTide technology developed at the University of Cardiff receiving European public funding in the 1990s, which is used in the GS-5734 compound structure.^27,57,58^

## Conclusion

 This case study of remdesivir discussed the collective, cumulative and uncertain characteristics of the innovation process and highlights the lack of transparency in our biomedical R&D system, the need for public investment in the innovation process, and the “time-lag” between risk-taking and reward. It further showed the inequity in public risk-taking and reward, resulting in rewards to few actors holding the key patent rights, while the return to the public in the form of equitable access and affordable pricing is limited. Beyond the necessity of treating remdesivir as a global public good, we argue that biomedical innovation needs to be viewed in the broader concept of public value creation. This requires further research on the setting of research priorities, the establishment of inclusive processes for decision-making and the development of a framework for public return emphasising health equity. Finally, with this study we aimed to create an analysis framework applicable to other health innovations relevant to the COVID-19 pandemic and new health technologies more generally.

## Acknowledgements

 The authors would like to thank Dzintars Gotham, Anna Peiris, Natalie Rhodes, and Rhiannon Osborne for comments on earlier versions of the manuscript.

## Ethical issues

 The data in this study were extracted from previously published academic articles and a publicly-accessibly trial registry and therefore did not require ethical approval.

## Competing interests

 Authors declare that they have no competing interests.

## Authors’ contributions

 Both authors contributed equally to the manuscript and responded to feedback and comments from colleagues.

## Disclaimers

 The authors of this paper are members of Universities Allied for Essential Medicines. However, views expressed in this paper are their own and not necessarily that of Universities Allied for Essential Medicines Europe.

## Authors’ affiliations

^1^University Medical Center Groningen, University of Groningen, Groningen, The Netherlands. ^2^Harvey E. Beardmore Division of Pediatric Surgery, The Montreal Children’s Hospital, McGill University Health Centre, Montreal, QC, Canada. ^3^Amsterdam UMC, University of Amsterdam, Amsterdam, the Netherlands. ^4^Department of Global Health and Development, London School of Hygiene and Tropical Medicine, London, UK.

## Supplementary files

Supplementary file 1. Identification of Primary Research Studies Analyzed for Author Affiliation and Grant Allocations.

Supplementary file 2. Author Affiliations and Grants for Identified Pre-pandemic Articles on Remdesivir.

Supplementary file 3. Clinical Trials on remdesivir Registered on ClinicalTrials.gov.

## References

[1] COVID-19 Dashboard by the Center for Systems Science and Engineering (CSSE) at Johns Hopkins University. Johns Hopkins University website. https://coronavirus.jhu.edu/map.html. Accessed July 22, 2020. Published 2020.

[2] Thanh Le T, Andreadakis Z, Kumar A (2020). The COVID-19 vaccine development landscape. Nat Rev Drug Discov.

[3] Tracking Public Investment in Global COVID-19 Research and Development. Universities Allied for Essential Medicines website. https://www.publicmeds4covid.org. Accessed July 22, 2020. Published 2020.

[4] Health Topics - Innovation. World Health Organization website. https://www.who.int/topics/innovation/en/. Accessed July 22, 2020.

[5] Webb Hooper M, Nápoles AM, Pérez-Stable EJ (2020). COVID-19 and racial/ethnic disparities. JAMA.

[6] Abrams EM, Szefler SJ (2020). COVID-19 and the impact of social determinants of health. Lancet Respir Med.

[7] Shadmi E, Chen Y, Dourado I (2020). Health equity and COVID-19: global perspectives. Int J Equity Health.

[8] Lazonick W, Mazzucato M (2013). The risk-reward nexus in the innovation-inequality relationship: who takes the risks?. who gets the rewards? Ind Corp Change.

[9] Health Topics - Health Equity. World Health Organization website. https://www.who.int/topics/health_equity/en/. Accessed July 22, 2020.

[10] Knowledge Ecology International (KEI). Role of the Federal Government in the Development of Remdesivir. https://www.keionline.org/wp-content/uploads/KEI-Briefing-Note-2020_1GS-5734-Remdesivir.pdf. Published 2020.

[11] Public Citizen. The Real Story of Remdesivir. https://www.citizen.org/wp-content/uploads/The-Real-Story-of-Remdesivir-final-May-7.pdf. Published 2020.

[12] Cho A, Saunders OL, Butler T (2012). Synthesis and antiviral activity of a series of 1’-substituted 4-aza-7,9-dideazaadenosine C-nucleosides. Bioorg Med Chem Lett.

[13] Warren TK, Jordan R, Lo MK (2016). Therapeutic efficacy of the small molecule GS-5734 against Ebola virus in rhesus monkeys. Nature.

[14] Jacobs M, Rodger A, Bell DJ (2016). Late Ebola virus relapse causing meningoencephalitis: a case report. Lancet.

[15] Sheahan TP, Sims AC, Graham RL (2017). Broad-spectrum antiviral GS-5734 inhibits both epidemic and zoonotic coronaviruses. Sci Transl Med.

[16] Lo MK, Jordan R, Arvey A (2017). GS-5734 and its parent nucleoside analog inhibit Filo-, Pneumo-, and Paramyxoviruses. Sci Rep.

[17] Dörnemann J, Burzio C, Ronsse A (2017). First newborn baby to receive experimental therapies survives Ebola virus disease. J Infect Dis.

[18] Siegel D, Hui HC, Doerffler E (2017). Discovery and synthesis of a phosphoramidate prodrug of a Pyrrolo[2,1-f][triazin-4-amino] adenine C-nucleoside (GS-5734) for the treatment of Ebola and emerging viruses. J Med Chem.

[19] Agostini ML, Andres EL, Sims AC (2018). Coronavirus susceptibility to the antiviral remdesivir (GS-5734) is mediated by the viral polymerase and the proofreading exoribonuclease. mBio.

[20] Jordan PC, Liu C, Raynaud P (2018). Initiation, extension, and termination of RNA synthesis by a paramyxovirus polymerase. PLoS Pathog.

[21] Lo MK, Feldmann F, Gary JM (2019). Remdesivir (GS-5734) protects African green monkeys from Nipah virus challenge. Sci Transl Med.

[22] Mulangu S, Dodd LE, Davey RT Jr (2019). A randomized, controlled trial of Ebola virus disease therapeutics. N Engl J Med.

[23] Tchesnokov EP, Feng JY, Porter DP, Götte M (2019). Mechanism of inhibition of Ebola virus RNA-dependent RNA polymerase by remdesivir. Viruses.

[24] McMullan LK, Flint M, Chakrabarti A (2019). Characterisation of infectious Ebola virus from the ongoing outbreak to guide response activities in the Democratic Republic of the Congo: a phylogenetic and in vitro analysis. Lancet Infect Dis.

[25] Brown AJ, Won JJ, Graham RL (2019). Broad spectrum antiviral remdesivir inhibits human endemic and zoonotic deltacoronaviruses with a highly divergent RNA dependent RNA polymerase. Antiviral Res.

[26] Koplon S. $37.5 Million Grant will Address Research of High-Priority Infections. University of Alabama at Birmingham website. https://www.uab.edu/news/health/item/10307-37-5-million-grant-will-address-research-of-high-priority-infections. Accessed July 22, 2020.

[27] Mehellou Y, Rattan HS, Balzarini J (2018). The ProTide prodrug technology: from the concept to the clinic. J Med Chem.

[28] Grein J, Ohmagari N, Shin D (2020). Compassionate use of remdesivir for patients with severe COVID-19. N Engl J Med.

[29] Biotechnology Innovation Association. Clinical Development Success Rates 2006-2015. https://www.bio.org/sites/default/files/legacy/bioorg/docs/Clinical%20Development%20Success%20Rates%202006-2015%20-%20BIO,%20Biomedtracker,%20Amplion%202016.pdf. Published 2016.

[30] Dyer O (2019). Two Ebola treatments halve deaths in trial in DRC outbreak. BMJ.

[31] Wang Y, Zhang D, Du G (2020). Remdesivir in adults with severe COVID-19: a randomised, double-blind, placebo-controlled, multicentre trial. Lancet.

[32] Beigel JH, Tomashek KM, Dodd LE (2020). Remdesivir for the treatment of COVID-19 - preliminary report. N Engl J Med.

[33] Coronavirus (COVID-19) Update: FDA Issues Emergency Use Authorization for Potential COVID-19 Treatment. U.S. Food and Drug Administration website. https://www.fda.gov/news-events/press-announcements/coronavirus-covid-19-update-fda-issues-emergency-use-authorization-potential-covid-19-treatment. Accessed July 22, 2020. Published 2020.

[34] Horby P, Lim WS, Emberson JR (2020). Dexamethasone in hospitalized patients with COVID-19 - preliminary report. N Engl J Med.

[35] Knowledge Ecology International (KEI). Other Transaction Agreements: Government Contracts That May Eliminate Protections for the Public on Pricing, Access and Competition, Including in Connection with COVID-19. KEI Briefing Note 2020:3 June 24, 2020. https://www.keionline.org/wp-content/uploads/KEI-Briefing-OTA-24June2020.pdf. Published 2020.

[36] Contract Data Reports for FPDS Users. U.S. General Services Administration Federal Government website. https://beta.sam.gov/awards/85565339%2BAWARD?keywords=W911QY1690001&sort=-relevance&index=&is_active=true&page=2. Accessed July 22, 2020.

[37] Galkina Cleary E, Beierlein JM, Khanuja NS, McNamee LM, Ledley FD (2018). Contribution of NIH funding to new drug approvals 2010-2016. Proc Natl Acad Sci U S A.

[38] Nayak RK, Avorn J, Kesselheim AS (2019). Public sector financial support for late stage discovery of new drugs in the United States: cohort study. BMJ.

[39] Chakma J, Sun GH, Steinberg JD, Sammut SM, Jagsi R (2014). Asia’s ascent--global trends in biomedical R&D expenditures. N Engl J Med.

[40] Association of American Universities (AAU). Basic Scientific and Engineering Research at U.S. Universities. AAU; 2015. https://www.aau.edu/node/10671. Accessed July 22, 2020.

[41] Young R, Bekele T, Gunn A (2018). Developing new health technologies for neglected diseases: a pipeline portfolio review and cost model. Gates Open Res.

[42] Azoulay P, Zivin JSG, Li D, Sampat BN. Public R&D Investments and Private-Sector Patenting: Evidence from NIH Funding Rules. https://www.nber.org/papers/w20889.pdf. Published 2015. 10.1093/restud/rdy034 PMC6818650 31662587

[43] Gilead Sciences Statement on Request to Rescind Remdesivir Orphan Drug Designation. Gilead Sciences website. https://www.gilead.com/news-and-press/company-statements/gilead-sciences-statement-on-request-to-rescind-remdesivir-orphan-drug-designation. Accessed July 22, 2020. Published 2020.

[44] Hill A, Wang J, Levi J, Heath K, Fortunak J (2020). Minimum costs to manufacture new treatments for COVID-19. J Virus Erad.

[45] Silverman E. Gilead Signs Licenses for Generic Companies to Make and Sell Remdesivir in 127 Countries. STAT. 2020. https://www.statnews.com/pharmalot/2020/05/12/gilead-generics-remdesivir-covid19-coronavirus-licenses/. Accessed July 22, 2020.

[46] Sternlicht A. Entire Stockpile of Coronavirus Treatment Remdesivir Donated to Government, Says CEO. Forbes. May 3, 2020. https://www.forbes.com/sites/alexandrasternlicht/2020/05/03/entire-stockpile-of-coronavirus-treatment-remdesivir-donated-to-government-says-ceo/#4e6b548725fb. Accessed July 22, 2020.

[47] An Open Letter from Daniel O’Day, Chairman & CEO, Gilead Sciences. Gilead Sciences website. https://www.gilead.com/news-and-press/press-room/press-releases/2020/6/an-open-letter-from-daniel-oday-chairman--ceo-gilead-sciences. Accessed July 22, 2020. Published 2020.

[48] Mitra AK, Cavale S. India’s Cipla Prices its Generic Remdesivir at $53.34 Per Vial, Below Rivals. Reuters. July 8, 2020. https://www.reuters.com/article/us-health-coronavirus-cipla/indias-cipla-prices-its-generic-remdesivir-at-53-34-per-vial-below-rivals-idUSKBN2492Q3. Accessed July 22, 2020.

[49] Trump Administration Secures New Supplies of Remdesivir for the United States. U.S. Department of Health & Human Services website. https://www.hhs.gov/about/news/2020/06/29/trump-administration-secures-new-supplies-remdesivir-united-states.html. Accessed July 22, 2020. Published 2020.

[50] World Health Organization Regional Office for South-East Asia. Equity in Access to Public Health, Report and Documentation of the Technical Discussions Held in Conjunction with the 37th Meeting of CCPDM New Delhi, August 31, 2000. New Delhi: WHO; 2000.

[51] Surgo Foundation. Bringing Greater Precision to the COVID-19 Response. Surgo Foundation; 2020.

[52] Jamison DT, Summers LH, Alleyne G (2013). Global health 2035: a world converging within a generation. Lancet.

[53] Moon S, Røttingen JA, Frenk J (2017). Global public goods for health: weaknesses and opportunities in the global health system. Health Econ Policy Law.

[54] Mazzucato M, Li HL, Darzi A (2020). Is it time to nationalise the pharmaceutical industry?. BMJ.

[55] Bozeman B (2020). Public value science. Issues Sci Technol.

[56] Sarewitz D (2020). The science policy we deserve. Issues Sci Technol.

[57] McGuigan C, Cahard D, Sheeka HM, De Clercq E, Balzarini J (1996). Aryl phosphoramidate derivatives of d4T have improved anti-HIV efficacy in tissue culture and may act by the generation of a novel intracellular metabolite. J Med Chem.

[58] Roy V. The Financialization of a Cure: A Political Economy of Biomedical Innovation, Pricing and Public Health. https://pdfs.semanticscholar.org/77c2/90878829e0e6d31d6dcc1b5a6b6fd132c449.pdf. Published 2017.

