# Supplementary material for: Public Risk-Taking and Rewards During the COVID-19 Pandemic - A Case Study of Remdesivir in the Context of Global Health Equity
Source: Int J Health Policy Manag. 2020 Sep 6;11(5):567–78. doi: 10.34172/ijhpm.2020.166 (PMC9309932; doi:10.34172/ijhpm.2020.166)
Supplement: Supplementary file 1 — Identification of Primary Research Studies Analyzed for Author Affiliation and Grant Allocations. [file ijhpm-11-567-s001.pdf]

Supplementary file 1. Identification of Primary Research Studies Analyzed for Author Affiliation and Grant Allocations

Inclusion criteria:

- Primary research study
- Investigates GS-5734 or Remdesivir (it is not just mentioned in the intro or discussion)

Exclusion criteria:

- Publication not in English
- Reviews/systematic reviews/meta-analyses
- Published after end of 2019

Identified articles:

1. Cho, A., Saunders, O. L., Butler, T., Zhang, L., Xu, J., Vela, J. E., Feng, J. Y., Ray, A. S., & Kim, C. U. (2012). Synthesis and antiviral activity of a series of 1'-substituted 4-aza-7,9-dideazaadenosine C-nucleosides. Bioorganic & medicinal chemistry letters, 22(8), 2705–2707. https://doi.org/10.1016/j.bmcl.2012.02.105
2. Agostini ML, Andres EL, Sims AC, et al. Coronavirus Susceptibility to the Antiviral Remdesivir (GS-5734) Is Mediated by the Viral Polymerase and the Proofreading Exoribonuclease. *mBio*. 2018;9(2):e00221-18. Published 2018 Mar 6. doi:10.1128/mBio.00221-18
3. Sheahan TP, Sims AC, Graham RL, et al. Broad-spectrum antiviral GS-5734 inhibits both epidemic and zoonotic coronaviruses. *Sci Transl Med*. 2017;9(396):eaal3653. doi:10.1126/scitranslmed.aal3653
4. Warren TK, Jordan R, Lo MK, et al. Therapeutic efficacy of the small molecule GS-5734 against Ebola virus in rhesus monkeys [published correction appears in ACS Chem Biol. 2016 May 20;11(5):1463]. *Nature*. 2016;531(7594):381-385. doi:10.1038/nature17180
5. Lo MK, Jordan R, Arvey A, et al. GS-5734 and its parent nucleoside analog inhibit Filo-, Pneumo-, and Paramyxoviruses. *Sci Rep*. 2017;7:43395. Published 2017 Mar 6. doi:10.1038/srep43395
6. Siegel D, Hui HC, Doerffler E, et al. Discovery and Synthesis of a Phosphoramidate Prodrug of a Pyrrolo[2,1-f][triazin-4-amino] Adenine C-Nucleoside (GS-5734) for the Treatment of Ebola and Emerging Viruses. *J Med Chem*. 2017;60(5):1648-1661. doi:10.1021/acs.jmedchem.6b01594
7. Tchesnokov EP, Feng JY, Porter DP, Götte M. Mechanism of Inhibition of Ebola Virus RNA-Dependent RNA Polymerase by Remdesivir. *Viruses*. 2019;11(4):326. Published 2019 Apr 4. doi:10.3390/v11040326
8. Mulangu S, Dodd LE, Davey RT Jr, et al. A Randomized, Controlled Trial of Ebola Virus Disease Therapeutics. *N Engl J Med*. 2019;381(24):2293-2303. doi:10.1056/NEJMoa1910993
9. Jacobs M, Rodger A, Bell DJ, et al. Late Ebola virus relapse causing meningoencephalitis: a case report. *Lancet*. 2016;388(10043):498-503. doi:10.1016/S0140-6736(16)30386-5
10. Lo MK, Feldmann F, Gary JM, et al. Remdesivir (GS-5734) protects African green monkeys from Nipah virus challenge. Sci Transl Med. 2019;11(494):eaau9242. doi:10.1126/scitranslmed.aau9242
11. Dörnemann J, Burzio C, Ronsse A, et al. First Newborn Baby to Receive Experimental Therapies Survives Ebola Virus Disease. *J Infect Dis*. 2017;215(2):171-174. doi:10.1093/infdis/jiw493
12. Jordan PC, Liu C, Raynaud P, et al. Initiation, extension, and termination of RNA synthesis by a paramyxovirus polymerase. *PLoS Pathog*. 2018;14(2):e1006889. Published 2018 Feb 9. doi:10.1371/journal.ppat.1006889
13. Brown AJ, Won JJ, Graham RL, et al. Broad spectrum antiviral remdesivir inhibits human endemic and zoonotic deltacoronaviruses with a highly divergent RNA dependent RNA polymerase. *Antiviral Res*. 2019;169:104541. doi:10.1016/j.antiviral.2019.104541
14. McMullan LK, Flint M, Chakrabarti A, et al. Characterisation of infectious Ebola virus from the ongoing outbreak to guide response activities in the Democratic Republic of the Congo: a phylogenetic and in vitro analysis. *Lancet Infect Dis*. 2019;19(9):1023-1032. doi:10.1016/S1473-3099(19)30291-9

PubMed Search (6/7/2020)

Search terms: Remdesivir OR GS-5734. Publications up to end of 2019.

Included

1. Agostini ML, Andres EL, Sims AC, et al. Coronavirus Susceptibility to the Antiviral Remdesivir (GS-5734) Is Mediated by the Viral Polymerase and the Proofreading Exoribonuclease. mBio. 2018;9(2):e00221-18. Published 2018 Mar 6. doi:10.1128/mBio.00221-18
2. Sheahan TP, Sims AC, Graham RL, et al. Broad-spectrum antiviral GS-5734 inhibits both epidemic and zoonotic coronaviruses. Sci Transl Med. 2017;9(396):eaal3653. doi:10.1126/scitranslmed.aal3653
3. Warren TK, Jordan R, Lo MK, et al. Therapeutic efficacy of the small molecule GS-5734 against Ebola virus in rhesus monkeys [published correction appears in ACS Chem Biol. 2016 May 20;11(5):1463]. Nature. 2016;531(7594):381-385. doi:10.1038/nature17180
4. Lo MK, Jordan R, Arvey A, et al. GS-5734 and its parent nucleoside analog inhibit Filo-, Pneumo-, and Paramyxoviruses. Sci Rep. 2017;7:43395. Published 2017 Mar 6. doi:10.1038/srep43395
5. Siegel D, Hui HC, Doerffler E, et al. Discovery and Synthesis of a Phosphoramidate Prodrug of a Pyrrolo[2,1-f]

[triazin-4-amino] Adenine C-Nucleoside (GS-5734) for the Treatment of Ebola and Emerging Viruses. J Med Chem. 2017;60(5):1648-1661. doi:10.1021/acs.jmedchem.6b01594
6. Tchesnokov EP, Feng JY, Porter DP, Götte M. Mechanism of Inhibition of Ebola Virus RNA-Dependent RNA Polymerase by Remdesivir. Viruses. 2019;11(4):326. Published 2019 Apr 4. doi:10.3390/v11040326
7. Mulangu S, Dodd LE, Davey RT Jr, et al. A Randomized, Controlled Trial of Ebola Virus Disease Therapeutics. N Engl J Med. 2019;381(24):2293-2303. doi:10.1056/NEJMoa1910993
8. Jacobs M, Rodger A, Bell DJ, et al. Late Ebola virus relapse causing meningoencephalitis: a case report. Lancet. 2016;388(10043):498-503. doi:10.1016/S0140-6736(16)30386-5
9. Lo MK, Feldmann F, Gary JM, et al. Remdesivir (GS-5734) protects African green monkeys from Nipah virus challenge. Sci Transl Med. 2019;11(494):eaau9242. doi:10.1126/scitranslmed.aau9242
10. Dörnemann J, Burzio C, Ronsse A, et al. First Newborn Baby to Receive Experimental Therapies Survives Ebola Virus Disease. J Infect Dis. 2017;215(2):171-174. doi:10.1093/infdis/jiw493
11. Jordan PC, Liu C, Raynaud P, et al. Initiation, extension, and termination of RNA synthesis by a paramyxovirus polymerase. PLoS Pathog. 2018;14(2):e1006889. Published 2018 Feb 9. doi:10.1371/journal.ppat.1006889
12. Brown AJ, Won JJ, Graham RL, et al. Broad spectrum antiviral remdesivir inhibits human endemic and zoonotic deltacoronaviruses with a highly divergent RNA dependent RNA polymerase. Antiviral Res. 2019;169:104541. doi:10.1016/j.antiviral.2019.104541
13. McMullan LK, Flint M, Chakrabarti A, et al. Characterisation of infectious Ebola virus from the ongoing outbreak to guide response activities in the Democratic Republic of the Congo: a phylogenetic and in vitro analysis. Lancet Infect Dis. 2019;19(9):1023-1032. doi:10.1016/S1473-3099(19)30291-9

Excluded

1. Remdesivir. In: *Drugs and Lactation Database (LactMed)*. Bethesda (MD): National Library of Medicine (US); 2006.
2. Cardile AP, Warren TK, Martins KA, Reisler RB, Bavari S. Will There Be a Cure for Ebola?. *Annu Rev Pharmacol Toxicol*. 2017;57:329-348. doi:10.1146/annurev-pharmtox-010716-105055
3. Beigel JH, Nam HH, Adams PL, et al. Advances in respiratory virus therapeutics - A meeting report from the 6th isirv Antiviral Group conference. *Antiviral Res*. 2019;167:45-67. doi:10.1016/j.antiviral.2019.04.006
4. Nakkazi E. Randomised controlled trial begins for Ebola therapeutics. *Lancet*. 2018;392(10162):2338. doi:10.1016/S0140-6736(18)33011-3
5. Check Hayden E. Experimental drugs poised for use in Ebola outbreak. *Nature*. 2018;557(7706):475-476. doi:10.1038/d41586-018-05205-x
6. Bixler SL, Duplantier AJ, Bavari S. Discovering Drugs for the Treatment of Ebola Virus. *Curr Treat Options Infect Dis*. 2017;9(3):299-317. doi:10.1007/s40506-017-0130-z
7. Inungu J, Iheduru-Anderson K, Odio OJ. Recurrent Ebolavirus disease in the Democratic Republic of Congo: update and challenges. AIMS Public Health. 2019;6(4):502-513. Published 2019 Nov 20. doi:10.3934/publichealth.2019.4.502
8. Public health round-up. *Bull World Health Organ*. 2019;97(1):4-5. doi:10.2471/BLT.19.010119
9. Madelain V, Baize S, Jacquot F, et al. Ebola viral dynamics in nonhuman primates provides insights into virus immuno-pathogenesis and antiviral strategies. *Nat Commun*. 2018;9(1):4013. Published 2018 Oct 1. doi:10.1038/s41467-018-06215-z
10. De Clercq E. New Nucleoside Analogues for the Treatment of Hemorrhagic Fever Virus Infections. *Chem Asian J*. 2019;14(22):3962-3968. doi:10.1002/asia.201900841
11. Gupta P, Goyal K, Kanta P, Ghosh A, Singh MP. Novel 2019-coronavirus on new year's Eve. *Indian J Med Microbiol*. 2019;37(4):459-477. doi:10.4103/ijmm.IJMM_20_54
12. Lucey DR. New treatments for Ebola virus disease. BMJ. 2019;366:l5371. Published 2019 Sep 6. doi:10.1136/bmj.l5371
13. Hoenen T, Groseth A, Feldmann H. Therapeutic strategies to target the Ebola virus life cycle. *Nat Rev Microbiol*. 2019;17(10):593-606. doi:10.1038/s41579-019-0233-2

Ovid Search (18/7/2020)

Date: 18/7/2020

Initial search returned 171 articles, however by applying a filter to identify articles only upt to end of 2019 and removing duplicated entries returned 21 articles only. After excluding reviews and articles already identified in PubMed, additional articles identified in bold (n=0).

Database: Embase <1980 to 2020 Week 28>, Global Health <1910 to 2020 Week 28>, Journals@Ovid Full Text <July 16, 2020>, Ovid MEDLINE(R) <1946 to July Week 2 2020>, LSHTM Journals@Ovid, Econlit <1886 to July 09, 2020>, Ovid MEDLINE(R) Epub Ahead of Print <July 16, 2020>, Social Policy and Practice <202004>

Included

1. Brown, A. J., Won, J. J., Graham, R. L., Iii, K. H. D., Sims, A. C., Feng JoyY., Cihlar, T., Denison, M. R., Baric, R. S., Sheahan, T. P., Broad spectrum antiviral remdesivir inhibits human endemic and zoonotic deltacoronaviruses with a highly divergent RNA dependent RNA polymerase.. Antiviral Research; 2019 169In: Global Health [Internet]. http://ovidsp.ovid.com/ovidweb.cgi?T=JS&PAGE=reference&D=cagh&NEWS=N&AN=20203253074

2. McMullan, L. K., Flint, M., Ayan Chakrabarti, Guerrero, L., Lo, M. K., Porter, D., Nichol, S. T., Spiropoulou, C. F., Albarino, C., Characterisation of infectious Ebola virus from the ongoing outbreak to guide response activities in the Democratic Republic of the Congo: a phylogenetic and in vitro analysis.. Lancet Infectious Diseases; 2019 19(9):1023-1032. In: Global Health [Internet]. http://ovidsp.ovid.com/ovidweb.cgi?T=JS&PAGE=reference&D=cagh&NEWS=N&AN=20193478732
3. Tchesnokov, E. P., Feng, J. Y., Porter, D. P., Gotte, M., Mechanism of inhibition of Ebola virus RNA-dependent RNA polymerase by remdesivir.. Viruses; 2019 11(4):326. In: Global Health [Internet]. http://ovidsp.ovid.com/ovidweb.cgi?T=JS&PAGE=reference&D=cagh&NEWS=N&AN=20193424780
4. Madelain, V., Baize, S., Jacquot, F., Reynard, S., Fizet, A., Barron, S., Solas, C., Lacarelle, B., Carbonnelle, C., Mentre, F., Raoul, H., Lamballerie, X. de, Guedj, J., Ebola viral dynamics in nonhuman primates provides insights into virus immuno-pathogenesis and antiviral strategies.. Nature Communications; 2018 9(10):4013. In: Global Health [Internet]. http://ovidsp.ovid.com/ovidweb.cgi?T=JS&PAGE=reference&D=cagh&NEWS=N&AN=20193317776
5. Siegel, D., Hui, H. C., Doerffler, E., Clarke, M. O., Chun Kwon, Zhang LiJun, Neville, S., Carra, E., Lew, W., Ross, B., Wang, Q., Wolfe, L., Jordan, R., Soloveva, V., Knox, J., Perry, J., Perron, M., Stray, K. M., Barauskas, O., Feng, J. Y., Xu Yili, Lee, G., Rheingold, A. L., Ray, A. S., Bannister, R., Strickley, R. [et al], Discovery and synthesis of a phosphoramidate prodrug of a pyrrolo[2,1-f][triazin-4-amino] adenine C-nucleoside (GS-5734) for the treatment of Ebola and emerging viruses.. Journal of Medicinal Chemistry; 2017 60(5):1648-1661. In: Global Health [Internet]. http://ovidsp.ovid.com/ovidweb.cgi?T=JS&PAGE=reference&D=cagh&NEWS=N&AN=20173197943
6. Agostini ML, Andres EL, Sims AC, Graham RL, Sheahan TP, Lu X, Smith EC, Case JB, Feng JY, Jordan R, Ray AS, Cihlar T, Siegel D, Mackman RL, Clarke MO, Baric RS, Denison MR. [ti] mBio [Internet]. 2018 [cited 2018 03 06];9(2):In: Ovid MEDLINE(R) Revisions [Internet].http://ovidsp.ovid.com/ovidweb.cgi?T=JS&PAGE=reference&D=medc&NEWS=N&AN=29511076
7. Mulangu S, Dodd LE, Davey RT Jr, Tshiani Mbaya O, Proschan M, Mukadi D, Lusakibanza Manzo M, Nzolo D, Tshomba Oloma A, Ibanda A, Ali R, Coulibaly S, Levine AC, Grais R, Diaz J, Lane HC, Muyembe-Tamfum JJ, PALM Writing Group, Sivahera B, Camara M, Kojan R, Walker R, Dighero-Kemp B, Cao H, Mukumbayi P, Mbala-Kingebeni P, Ahuka S, Albert S, Bonnett T, Crozier I, Duvenhage M, Proffitt C, Teitelbaum M, Moench T, Aboulhab J, Barrett K, Cahill K, Cone K, Eckes R, Hensley L, Herpin B, Higgs E, Ledgerwood J, Pierson J, Smolskis M, Sow Y, Tierney J, Sivapalasingam S, Holman W, Gettinger N, Vallee D, Nordwall J, PALM Consortium Study Team. [ti] N Engl J Med [Internet]. 2019 [cited 2019 12 12];381(24):2293-2303. In: Ovid MEDLINE(R) Revisions [Internet].http://ovidsp.ovid.com/ovidweb.cgi?T=JS&PAGE=reference&D=medc&NEWS=N&AN=31774950
8. Sheahan TP, Sims AC, Graham RL, Menachery VD, Gralinski LE, Case JB, Leist SR, Pyrc K, Feng JY, Trantcheva I, Bannister R, Park Y, Babusis D, Clarke MO, Mackman RL, Spahn JE, Palmiotti CA, Siegel D, Ray AS, Cihlar T, Jordan R, Denison MR, Baric RS. [ti] Sci Transl Med [Internet]. 2017 [cited 2017 06 28];9(396):In: Ovid MEDLINE(R) Revisions [Internet]. http://ovidsp.ovid.com/ovidweb.cgi?T=JS&PAGE=reference&D=medc&NEWS=N&AN=28659436
9. Lo MK, Jordan R, Arvey A, Sudhamsu J, Shrivastava-Ranjan P, Hotard AL, Flint M, McMullan LK, Siegel D, Clarke MO, Mackman RL, Hui HC, Perron M, Ray AS, Cihlar T, Nichol ST, Spiropoulou CF. [ti] Sci. rep. [Internet]. 2017 [cited 2017 03 06];7:43395. In: Ovid MEDLINE(R) Revisions [Internet]. http://ovidsp.ovid.com/ovidweb.cgi?T=JS&PAGE=reference&D=medc&NEWS=N&AN=28262699
10. Dornemann J, Burzio C, Ronsse A, Sprecher A, De Clerck H, Van Herp M, Kolie MC, Yosifiva V, Caluwaerts S, McElroy AK, Antierens A. [ti] J Infect Dis [Internet]. 2017 [cited 2017 Jan 15];215(2):171-174. In: Ovid MEDLINE(R) Revisions [Internet]. http://ovidsp.ovid.com/ovidweb.cgi?T=JS&PAGE=reference&D=medc&NEWS=N&AN=28073857
11. Jacobs M, Rodger A, Bell DJ, Bhagani S, Cropley I, Filipe A, Gifford RJ, Hopkins S, Hughes J, Jabeen F, Johannessen I, Karageorgopoulos D, Lackenby A, Lester R, Liu RS, MacConnachie A, Mahungu T, Martin D, Marshall N, Mepham S, Orton R, Palmarini M, Patel M, Perry C, Peters SE, Porter D, Ritchie D, Ritchie ND, Seaton RA, Sreenu VB, Templeton K, Warren S, Wilkie GS, Zambon M, Gopal R, Thomson EC. [ti] Lancet [Internet]. 2016 [cited 2016 Jul 30];388(10043):498-503. In: Ovid MEDLINE(R) Revisions [Internet]. http://ovidsp.ovid.com/ovidweb.cgi?T=JS&PAGE=reference&D=medc&NEWS=N&AN=27209148
12. Warren TK, Jordan R, Lo MK, Ray AS, Mackman RL, Soloveva V, Siegel D, Perron M, Bannister R, Hui HC, Larson N, Strickley R, Wells J, Stuthman KS, Van Tongeren SA, Garza NL, Donnelly G, Shurtleff AC, Retterer CJ, Gharaibeh D, Zamani R, Kenny T, Eaton BP, Grimes E, Welch LS, Gomba L, Wilhelmsen CL, Nichols DK, Nuss JE, Nagle ER, Kugelman JR, Palacios G, Doerffler E, Neville S, Carra E, Clarke MO, Zhang L, Lew W, Ross B, Wang Q, Chun K, Wolfe L, Babusis D, Park Y, Stray KM, Trancheva I, Feng JY, Barauskas O, Xu Y, Wong P, Braun MR, Flint M, McMullan LK, Chen SS, Fearns R, Swaminathan S, Mayers DL, Spiropoulou CF, Lee WA, Nichol ST, Cihlar T, Bavari S. [ti] Nature [Internet]. 2016 [cited 2016 Mar 17];531(7594):381-5. In: Ovid MEDLINE(R) Revisions [Internet].http://ovidsp.ovid.com/ovidweb.cgi?T=JS&PAGE=reference&D=medc&NEWS=N&AN=26934220
13. Jordan PC, Liu C, Raynaud P, Lo MK, Spiropoulou CF, Symons JA, Beigelman L, Deval J. [ti] PLoS Pathog [Internet]. 2018 [cited 2018 02];14(2):e1006889. In: Ovid MEDLINE(R) [Internet]. http://ovidsp.ovid.com/ovidweb.cgi?T=JS&PAGE=reference&D=med15&NEWS=N&AN=29425244

Excluded

1. Bonovas S., Piovani D., Pansieri C., Peyrin-Biroulet L., Danese S. A snapshot of the ongoing clinical research on COVID-19. F1000 Res. [Internet]. 2019 8 no pagination. In: Embase Available from http://ovidsp.ovid.com/ovidweb.cgi?T=JS&PAGE=reference&D=emexb&NEWS=N&AN=631991136
2. Parakriti Gupta, Kapil Goyal, Poonam Kanta, Arnab Ghosh, Singh, M. P., Novel 2019-coronavirus on new year's eve.. Indian Journal of Medical Microbiology; 2019 37(4):459-477. In: Global Health [Internet]. http://ovidsp.ovid.com/ovidweb.cgi?T=JS&PAGE=reference&D=cagh&NEWS=N&AN=20203256718
3. Beigel, J. H., Nam, H. H., Adams, P. L., Krafft, A., Ince, W. L., El-Kamary, S. S., Sims, A. C., Advances in respiratory virus

therapeutics - a meeting report from the 6th isirv Antiviral Group conference.. Antiviral Research; 2019 167:45-67. In: Global Health [Internet]. http://ovidsp.ovid.com/ovidweb.cgi?T=JS&PAGE=reference&D=cagh&NEWS=N&AN=20203253051
4. Hoenen, T., Groseth, A., Feldmann, H., Therapeutic strategies to target the Ebola virus life cycle.. Nature Reviews Microbiology; 2019 17(10):593-606. In: Global Health [Internet]. http://ovidsp.ovid.com/ovidweb.cgi?T=JS&PAGE=reference&D=cagh&NEWS=N&AN=20193493562
5. Cardile, A. P., Warren, T. K., Martins, K. A., Reisler, R. B., Bavari, S., Will there be a cure for Ebola?. Annual Review of Pharmacology and Toxicology; 2017 57:329-348. In: Global Health [Internet]. http://ovidsp.ovid.com/ovidweb.cgi?T=JS&PAGE=reference&D=cagh&NEWS=N&AN=20183307525
6. De Clercq E. [ti] Chem Asian J [Internet]. 2019 [cited 2019 Nov 18];14(22):3962-3968. In: Ovid MEDLINE(R) Revisions [Internet]. http://ovidsp.ovid.com/ovidweb.cgi?T=JS&PAGE=reference&D=medc&NEWS=N&AN=31389664
7. Nakkazi E. [ti] Lancet [Internet]. 2018 [cited 2018 12 01];392(10162):2338. In: Ovid MEDLINE(R) Revisions [Internet]. http://ovidsp.ovid.com/ovidweb.cgi?T=JS&PAGE=reference&D=medc&NEWS=N&AN=30527603
8. Cardile AP, Warren TK, Martins KA, Reisler RB, Bavari S. [ti] Annu Rev Pharmacol Toxicol [Internet]. 2017 [cited 2017 01 06];57:329-348. In: Ovid MEDLINE(R) Revisions [Internet]. http://ovidsp.ovid.com/ovidweb.cgi?T=JS&PAGE=reference&D=medc&NEWS=N&AN=27959624

Google Search (18/7/2020)
A search of Google Scholar was conducted to identify any articles that were missed out upon within the PubMed and Ovid search. Search terms were “Remdesivir” OR “GS-5734” and a date filter for articles up to the end of 2019 was applied.
The first 100 articles were screened for relevance and reference snowballing was applied to the bibliography. 1 new study was identified next the 13 listed above.

1. Cho, A., Saunders, O. L., Butler, T., Zhang, L., Xu, J., Vela, J. E., Feng, J. Y., Ray, A. S., & Kim, C. U. (2012). Synthesis and antiviral activity of a series of 1’-substituted 4-aza-7,9-dideazaadenosine C-nucleosides. Bioorganic & medicinal chemistry letters, 22(8), 2705–2707. https://doi.org/10.1016/j.bmcl.2012.02.105
